# Analysis and identification of oxidative stress-ferroptosis related biomarkers in ischemic stroke

**DOI:** 10.1038/s41598-024-54555-2

**Published:** 2024-02-15

**Authors:** Lin-Ming Zhang, Xing-ling Liang, Gui-fei Xiong, xuan-lin Xing, Qiu-juan Zhang, Bing-ran Zhang, Ming-wei Liu

**Affiliations:** 1 Department of Neurology, The First Affiliated Hospital of Kunming Medical University, Kunming, 650032 Yunnan China; 2 Department of Emergency, The First Affiliated Hospital of Kunming Medical University, Kunming, 650032 Yunnan China; 3 Department of Emergency, People’s Hospital of Dali Bai Autonomous Prefecture, No. 35 Renmin South Road, Xiaguan Street, Dalí, 671000 Yunnan China

**Keywords:** Oxidative stress, Ischemic stroke, Ferroptosis, Biomarkers, Bioinformatics analysis, Cell biology, Genetics, Immunology, Molecular biology, Neuroscience, Biomarkers, Diseases, Molecular medicine, Neurology

## Abstract

Studies have shown that a series of molecular events caused by oxidative stress is associated with ferroptosis and oxidation after ischemic stroke (IS). Differential analysis was performed to identify differentially expressed mRNA (DEmRNAs) between IS and control groups. Critical module genes were identified using weighted gene co-expression network analysis (WGCNA). DEmRNAs, critical module genes, oxidative stress-related genes (ORGs), and ferroptosis-related genes (FRGs) were crossed to screen for intersection mRNAs. Candidate mRNAs were screened based on the protein–protein interaction (PPI) network and the MCODE plug-in. Biomarkers were identified based on two types of machine learning algorithms, and the intersection was obtained. Functional items and related pathways of the biomarkers were identified using gene set enrichment analysis (GSEA). Finally, single-sample GSEA (ssGSEA) and Wilcoxon tests were used to identify differential immune cells. An miRNA-mRNA-TF network was created. Quantitative real-time polymerase chain reaction (qRT-PCR) was performed to verify the expression levels of biomarkers in the IS and control groups. There were 8287 DE mRNAs between the IS and control groups. The genes in the turquoise module were selected as critical module genes for IS. Thirty intersecting mRNAs were screened for overlaps. Seventeen candidate mRNAs were also identified. Four biomarkers (CDKN1A, GPX4, PRDX1, and PRDX6) were identified using two types of machine-learning algorithms. GSEA results indicated that the biomarkers were associated with steroid biosynthesis. Nine types of immune cells (activated B cells and neutrophils) were markedly different between the IS and control groups. We identified 3747 miRNA-mRNA-TF regulatory pairs in the miRNA-mRNA-TF regulatory network, including hsa-miR-4469-CDKN1A-BACH2 and hsa-miR-188-3p-GPX4-ATF2. CDKN1A, PRDX1, and PRDX6 were upregulated in IS samples compared with control samples. This study suggests that four biomarkers (CDKN1A, GPX4, PRDX1, and PRDX6) are significantly associated with IS. This study provides a new reference for the diagnosis and treatment of IS.

## Introduction

Ischemic stroke (IS) is a disease caused by the interaction of multiple environmental and genetic risk factors and is the most common cause of disability^1^. In contrast, IS is caused by reduced blood supply in certain areas of the brain owing to vascular obstruction^2^. IS can cause a series of complex neuropathological and physiological events (including excitotoxicity, oxidative stress, neuroinflammation, and apoptosis, etc.) leading to brain injury^3^. Therefore, if the disease cannot be effectively prevented or slowed, it will become a serious public health problem. Currently, the routine treatment for IS is a tissue plasminogen activator (tPA) for intravenous thrombolysis. However, owing to the narrow therapeutic window and possibility of severe bleeding, the benefit range for patients is small^4^. Biomarker-based IS assessments may help predict diagnosis and determine the treatment direction for each patient.

Oxidative stress (OS) is an important factor in neuroinflammation and neuronal necrosis in IS and is mainly caused by an imbalance between the production and consumption of reactive oxygen species (ROS). Excess ROS can induce lipid peroxidation and the oxidation of proteins, DNA, and RNA, leading to neuronal dysfunction and death^5^. Ferroptosis is an iron-dependent oxidative stress-induced cell death pathway that plays an important role in IS^6^. In animal cells, it has been confirmed that mitochondrial oxidative stress regulates ferroptosis through the NRF2-ARE pathway^7^. In addition, a series of molecular events due to oxidative stress have been shown to be associated with ferroptosis and oxidation processes after the onset of IS, and to have common molecular targets such as lipid peroxidation and GSH depletion^8,9^. Therefore, exploring the role of oxidative stress- and ferroptosis-related genes in the pathogenesis of IS is helpful for further understanding its pathophysiology. More importantly, the discovery of biomarkers associated with oxidative stress and ferroptosis in IS may provide new clues for diagnosis and treatment.

Based on data from the Gene Expression Omnibus (GEO) public database, this study aimed to explore biomarkers related to oxidative stress-ferroptosis in IS, using a series of bioinformatics methods to lay a theoretical foundation for the diagnosis and treatment of IS.

## Methods

### Data sources

The IS-related datasets of blood samples in this study were acquired from the GEO database (http://www.ncbi.nlm.nih.gov/geo/). Based on previous research^10^, there were 15 samples (five control and 10 IS samples) in the GSE122709 dataset using the Empirical Bayes method, which was utilized as a training set. The validation set GSE140275 contained three control samples and three IS samples. In addition, 1399 oxidative stress-related genes (ORGs) and 259 ferroptosis-related genes (FRGs) were identified based on published literatures^11,12^.

### Differential expression analysis and weighted gene co-expression network analysis (WGCNA)

In our study, differentially expressed mRNA (DEmRNAs) between the IS and control groups in the GSE122709 dataset were acquired using the limma-trend method after processing the data using the edgeR (v 3.36.0)^13^ package (adj.p.value < 0.05, |log_2_FC|> 0.5). Heat and volcano maps of DE mRNA between the IS and control groups were plotted using the p heat map (v 0.7.7) (14 and ggplot2 (v 3.3.2)^15^ packages, respectively. WGCNA was performed on all the samples in the GSE122709 dataset to screen for critical modules. First, outlier samples were eliminated to ensure precision of the analysis using sample clustering. An appropriate soft threshold (β) was selected, such that the engagement between genes conformed to a scale-free distribution to the maximum extent. Then, according to the standard hybrid dynamic tree cutting algorithm, the minimum number of genes per gene module was set to 30, and MEDissThres was set to 0.2 to merge similar modules (the similarity correlation value was 0.8). Subsequently, correlation analysis was used to evaluate the relationships between modules and traits (IS and control groups). Finally, the genes in the module most relevant to IS were defined as critical module genes (hub mRNAs).

### The acquisition of candidate mRNAs

The above DEmRNAs, Hub mRNAs in the critical module, 1399 ORGs, and 259 FRGs were crossed to screen the intersection mRNAs. Furthermore, to investigate the related biological functions and pathways of the intersection mRNAs, Gene Ontology (GO) and Kyoto Encyclopedia of Genes and Genomes (KEGG)^16–18^ enrichment analyses were performed using the clusterProfiler (v 3.14.3) package^19^ (adj.p < 0.05). In addition, to explore whether there was an interaction among these intersecting mRNAs, we used the STRING online database (https://string-db.org/) to create a protein–protein interaction (PPI) network (confidence = 0.4). The MCODE plug-in was used to identify the key submodules of the PPI network, and mRNAs in the submodules were treated as candidate mRNAs.

### Identification and verification of biomarkers

Based on the above candidate mRNAs, least absolute shrinkage and selection operator (LASSO) and Support Vector Machine-Recursive Feature Elimination (SVM-RFE) algorithms were implemented to acquire feature mRNAs. The LASSO algorithm was performed using the glmnet (v 4.0–2) package^20^(family = ‘binomial,’ type.measure = ‘class,’ and nfold = 10). The SVM-RFE algorithm^21^ was performed using the E1071 (v 1.7-9) package^22^ (five-fold cross validation). Furthermore, the feature mRNAs acquired using the LASSO algorithm and those identified using the SVM-RFE algorithm were crossed to screen for biomarkers. In addition, to evaluate the diagnostic ability of biomarkers for IS, receiver operating characteristic (ROC) curves of these biomarkers were plotted in the GSE122709 (training set) and GSE140275 (validation set) datasets. Finally, the expression levels of the biomarkers were extracted from the training set GSE122709 and validation set GSE140275, and the expression levels of the above biomarkers between the IS and control groups were compared using the ggplot2 (v. 3.3.2) package^14^.

### Enrichment analysis and immune infiltration analysis

To further understand the related biological functions and involved signaling pathways of the biomarkers, gene set enrichment analysis (GSEA) was implemented based on the KEGG pathway gene set using GSEA Software (v 4.0.3)^23^, with a significance threshold of |NES|> 1 and NOM p.val < 0.05. Subsequently, to evaluate the degree of immune cell infiltration, the ssGSEA algorithm was used to analyze the infiltration abundance of 28 immune cells in all samples in the training set. Differential immune cells between IS and control samples were identified using the Wilcoxon test. The relationships between the biomarkers and differential immune cells were computed using Spearman’s method.

### The construction of regulatory network and drug prediction

Subsequently, miRNAs of these biomarkers were predicted based on the miRNet database (https://www.mirnet.ca/miRNet/home.xhtml) and miRDB online database (https://mirdb.org/). The human transcription factor target (hTFtarget) online database (http://bioinfo.life.hust.edu.cn/hTFtarget#!/) was used to predict the transcription factors (TFs) of the biomarkers. Finally, the miRNAs and TFs regulated by the same mRNA were screened and an miRNA-mRNA-TF network was created. In addition, the IC_50_ of chemical drugs as biomarkers for IS patients was predicted according to the gene expression profile and cell line expression profile in the Genomics of Drug Sensitivity in Cancer (GDSC) online database (https://www.cancerrxgene.org/). The Wilcoxon test was used to compute the differences in drug sensitivity between the IS and control groups.

### Quantitative real-time PCR (qRT-PCR) verification

Blood samples were obtained from patients with IS and control subjects, with written informed consent for participation in the study. This study was approved by the Ethics Committee of the First Affiliated Hospital of Kunming Medical University. Twenty pairs of blood samples were divided into two groups: 10 IS and 10 control. Total RNA was isolated from the samples and purified using TRIzol reagent (Ambion, Austin, Texas, U.S.) following the manufacturer’s instructions. The concentration of extracted RNA was measured using a nanophotometer (N50). Next, reverse transcription via SureScript-First-strand-cDNA-synthesis-kit (Servicebio, Wuhan, China) by an ordinary PCR instrument. The reverse transcription product cDNA was diluted 5–20 times with ddH2O (RNase/DNase-free). Polymerase chain reaction (PCR) amplification was performed on a CFX96 real-time quantitative PCR instrument. The samples were denatured at 95 °C for (pre-denaturation), followed by denaturation at 95 °C for 20 s (denaturation), annealing at 55 °C for 20 s (annealing), and elongation at 72 °C for 30 s (elongation). The reactions were subjected to 40 cycles. The primer sequences used are listed in Table 1.

**Table 1:** The primer sequences of biomarkers.

Primer	Sequence
CDKN1A F	GCACGGAAGGACTTTGTAAGG
CDKN1A R	CGGCGTTTGGAGTGGTAGAA
GPX4 F	CCTTTGCCGCCTACTGAAG
GPX4 R	GGTCGACGAGCTGAGTGTAG
PRDX1 F	TTCTTGCCTGTTGCCTCTTCC
PRDX1 R	GGTTCAACCAGGTTCCCGCA
PRDX6 F	GCTTCTTCGCCAGAACCAAC
PRDX6 R	GACGGTGGTATTGGCCTCAA
Internal reference-GAPDH F	CGAAGGTGGAGTCAACGGATTT
Internal reference-GAPDH R	ATGGGTGGAATCATATTGGAAC

### Ethics approval and consent to participate

This study conformed to the ethical guidelines of the Science Foundation of the National Natural Science Foundation of China. Written informed consent was obtained from the individual(s) and minor(s) legal guardian/next of kin for publication of any potentially identifiable images or data included in this study.

### Consent for publication

Written informed consent for the publication of clinical details and/or images was obtained from the patient/parent/guardian/relative of the patient.

## Results

### Acquisition of DEmRNAs and key modules

**Figure 1:**
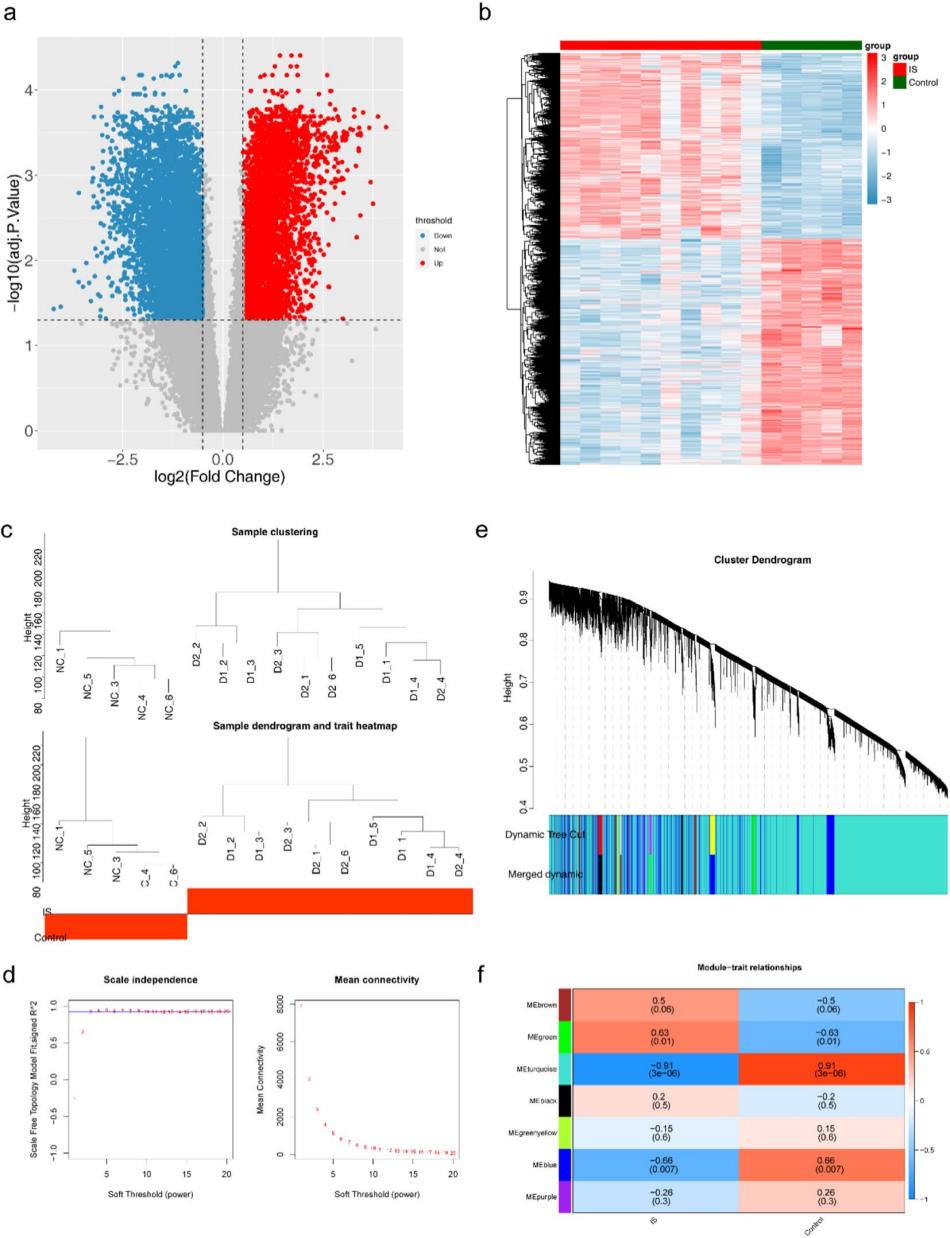
Acquisition of DEmRNAs and key modules. (**a**) Volcano map of mRNAs expression between groups. (**b**) Heat map of mRNA expression between groups. (**c**) Cluster analysis of dataset samples and Data Sample Clustering and Phenotypic Information. (**d**) Scale free soft threshold distribution. (**e**) Module Clustering Tree. (**f**) Heat map of correlation between modules and clinical traits.

In the GSE122709 dataset, there were 8287 DEmRNAs between the IS and control groups (Fig. 1a). The expression heat map of IS-associated DE mRNAs in the GSE122709 dataset is shown in Fig. 1b. Subsequently, WGCNA was used to select the critical module genes for clinical traits (IS and control). To ensure the accuracy of the analysis, we clustered samples to eliminate outliers. The results indicate that there were no outlier samples in the GSE122709 dataset. Thus, all the samples were used for subsequent analyses (Fig. 1c). In addition, when the soft threshold was three, the network was closest to the distribution without the network scale (Fig. 1d). Seven modules were obtained after the merging (Fig. 1e). The turquoise module (|Cor|= 0.91 and p < 0.05) was highly relevant to IS and control, and was therefore selected as the critical module. Therefore, the turquoise module contained 17799 Hub mRNAs was used for subsequent analyses (Fig. 1f).

### 17 candidate mRNAs were identified

**Figure 2:**
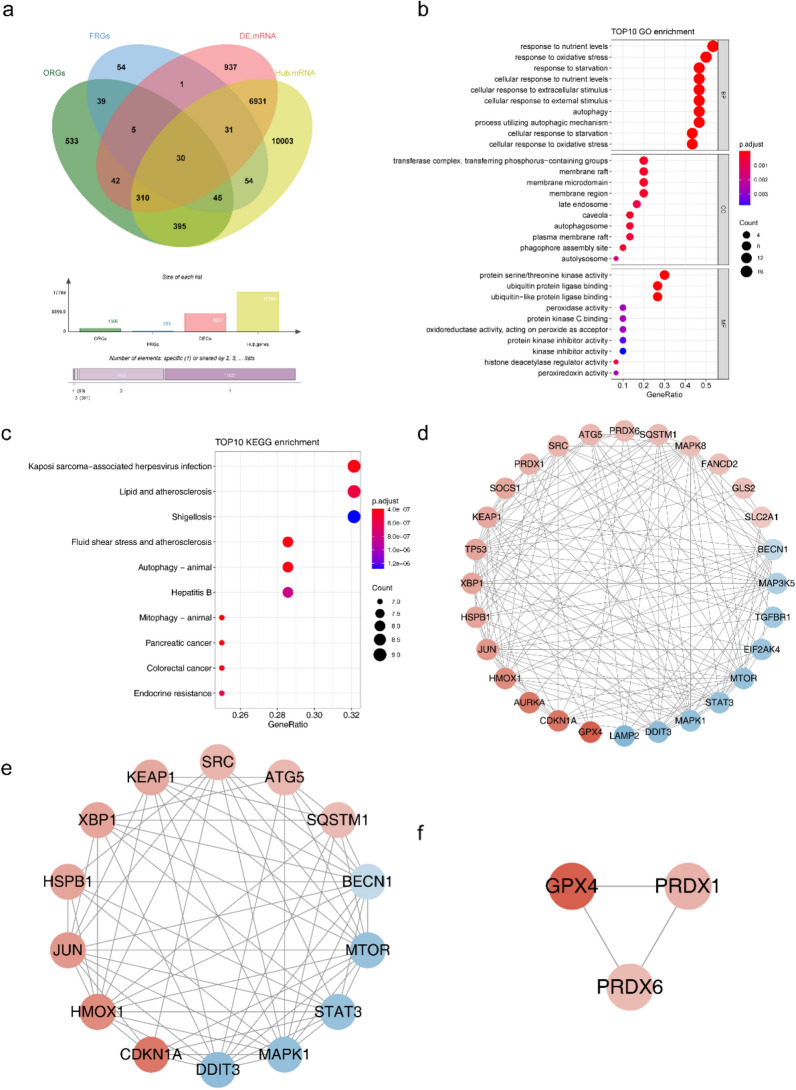
Identification of IS-related DEmRNAs and functional enrichment. (**a**) Venn diagram of intersection mRNA. (**b**) Bubble plot of intersection mRNA GO TOP10 enrichment results. (**c**) Bubble plot of intersection mRNA KEGG TOP10 enrichment results. (**d**) Protein Interaction Network Diagram. (**e**, **f**) Key sub modules in PPI network.

There were 30 intersection mRNAs overlapping 8287 DE mRNAs, 17799 hub mRNAs in the critical module, 1399 ORGs, and 259 FRGs (Fig. 2a). According to the GO functional enrichment analysis, intersecting mRNAs mainly participated in cellular responses to oxidative stress, cellular responses to extracellular stimuli, and responses to oxidative stress (Fig. 2b, Supplementary Table 1). KEGG functional enrichment analysis revealed that the intersecting mRNAs were associated with lipid and atherosclerosis, fluid shear stress, and atherosclerosis (Fig. 2c, Supplementary Table 2). Additionally, 154 protein interaction pairs were obtained from the PPI network, including 28 nodes. Moreover, MAPK8 showed higher connectivity in the PPI network (Fig. 2d). Seventeen candidate mRNAs were identified using the two key submodules. Cluster 1 had 14 candidate mRNAs, including BECN1, MTOR, STAT3, MAPK1, DDIT3, CDKN1A, HMOX1, JUN, HSPB1, XBP1, KEAP1, SRC, ATG5, and SQSTM1, and cluster 2 contained three candidate mRNAs (GPX4, PRDX1, and PRDX6) (Fig. 2e–f).

### Four biomarkers were screened out

**Figure 3:**
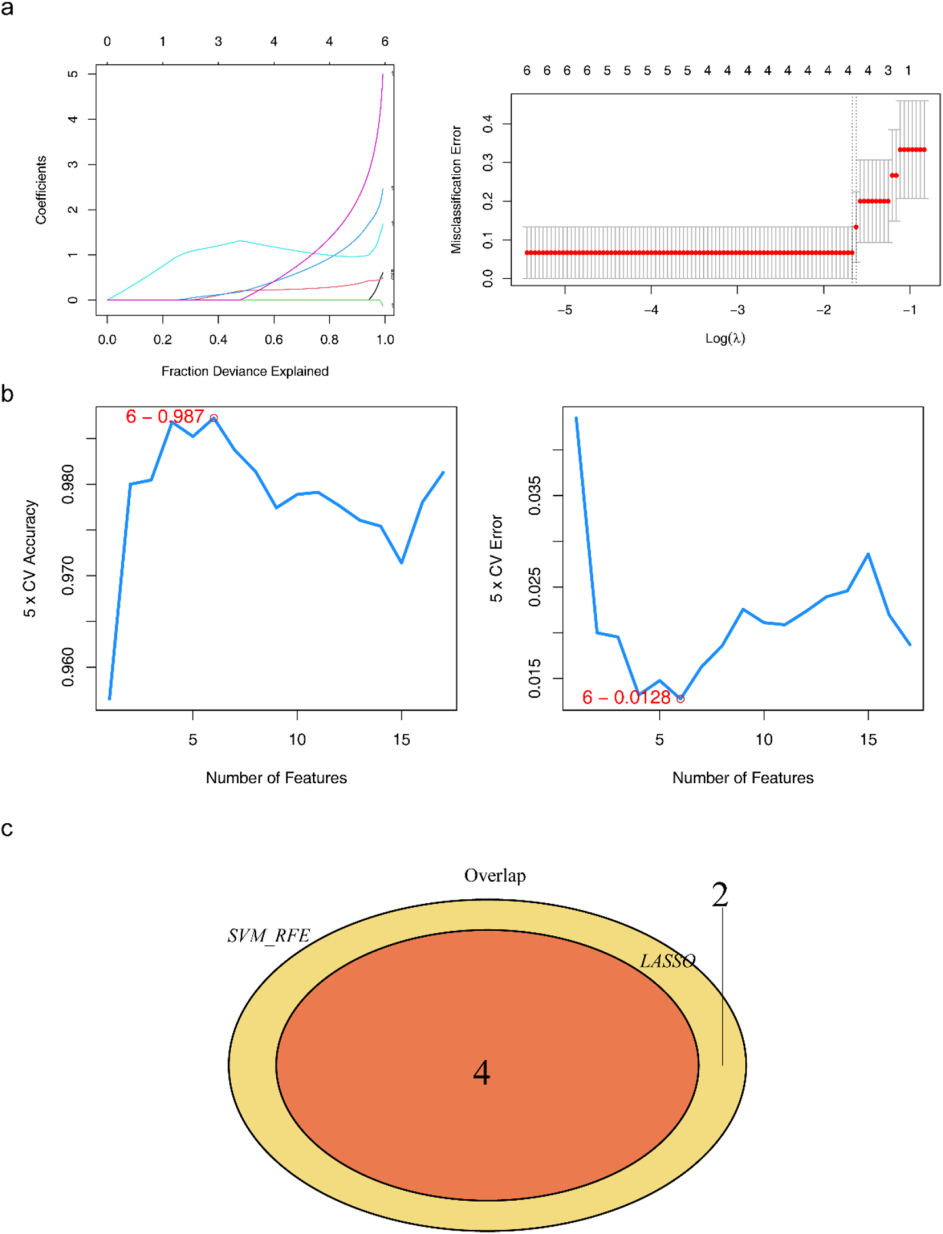
Machine learning for screening biomarkers. (**a**) Changes in mRNA coefficients in the LASSO model and LASSO Logic Coefficient Penalty Graph. (**b**) Support Vector Machine Model Accuracy (Left) and Error Rate (Right). (**c**) Venn diagram of biomarkers.

**Figure 4:**
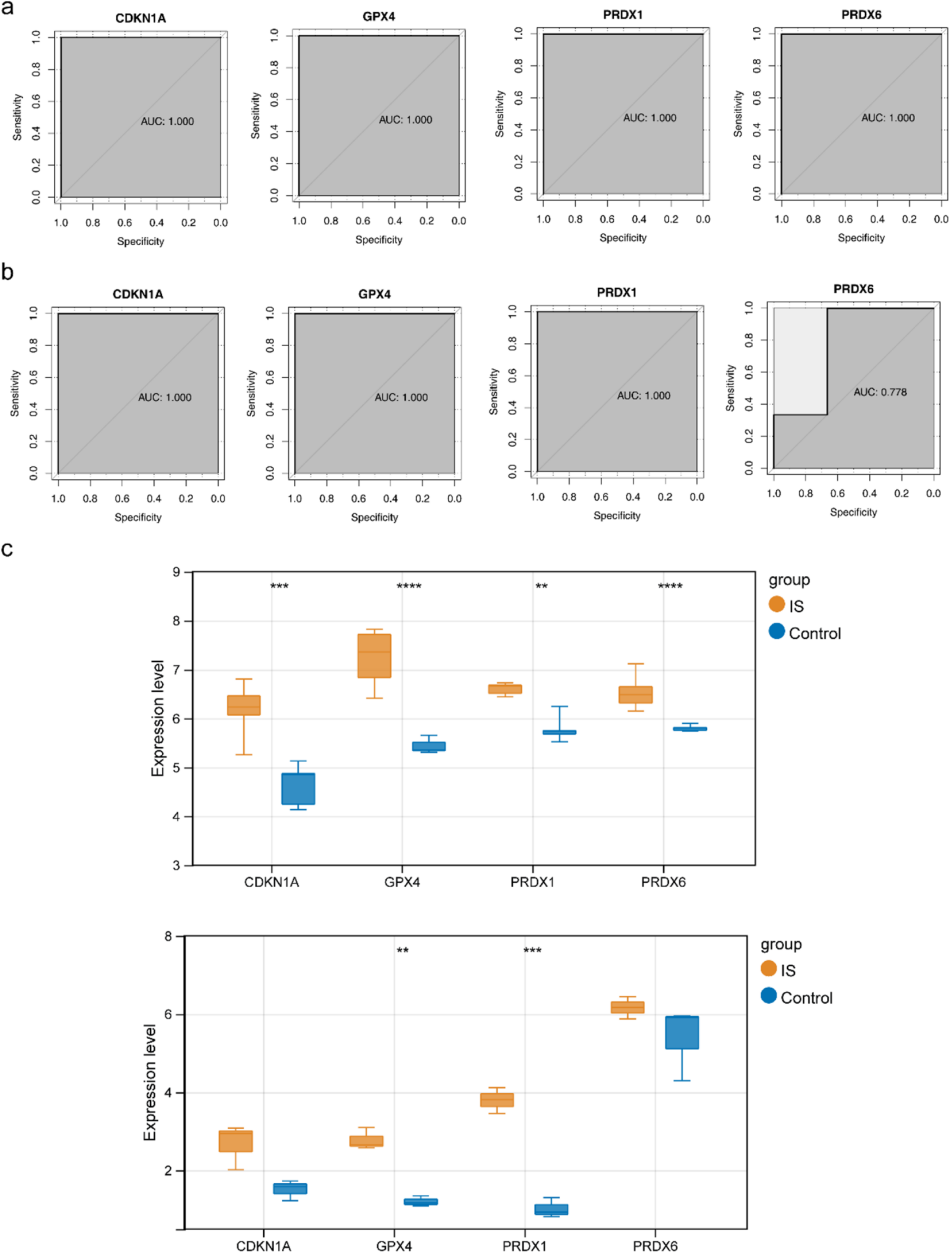
Analysis of biomarker expression and diagnostic value. (**a**) ROC curve of biomarkers (CDKN1A, GPX4, PRDX1, and PRDX6) in the training set. (**b**) ROC curves of Validation for concentrated biomarkers (CDKN1A, GPX4, PRDX1, and PRDX6). (**c**) Expression box diagram of biomarkers (CDKN1A, GPX4, PRDX1, and PRDX6) in the training set and expression box diagram for verifying the expression of concentrated biomarkers (CDKN1A, GPX4, PRDX1, and PRDX6).

In total, four mRNAs, CDKN1A, GPX4, PRDX1, and PRDX6, were identified using the LASSO algorithm (Fig. 3a). Six mRNAs (PRDX6, GPX4, PRDX1, CDKN1A, XBP1, and HMOX1) were identified using the SVM-RFE algorithm (Fig. 3b). Therefore, four biomarkers were screened by overlapping CDKN1A, GPX4, PRDX1, and PRDX6 (Fig. 3c). Furthermore, in the training set GSE122709, the area under the curve (AUC) values of the four biomarkers were all above 0.9, indicating that the biomarkers had the diagnostic ability to distinguish between the IS and control samples (Fig. 4a). In the validation set GSE140275, the AUC value of each biomarker was above 0.75, further verifying the diagnostic value of the biomarkers (Fig. 4b). Additionally, the expression levels of GPX4 and PRDX1 in the IS and control groups were significantly different between the training and validation sets. Although CDKN1A and PRDX6 levels did not differ in the validation set, the expression trend was consistent with that in the training set, and the four biomarkers showed an upward trend in the IS samples (Fig. 4c).

### GSEA of the biomarkers and the ssGSEA

**Figure 5:**
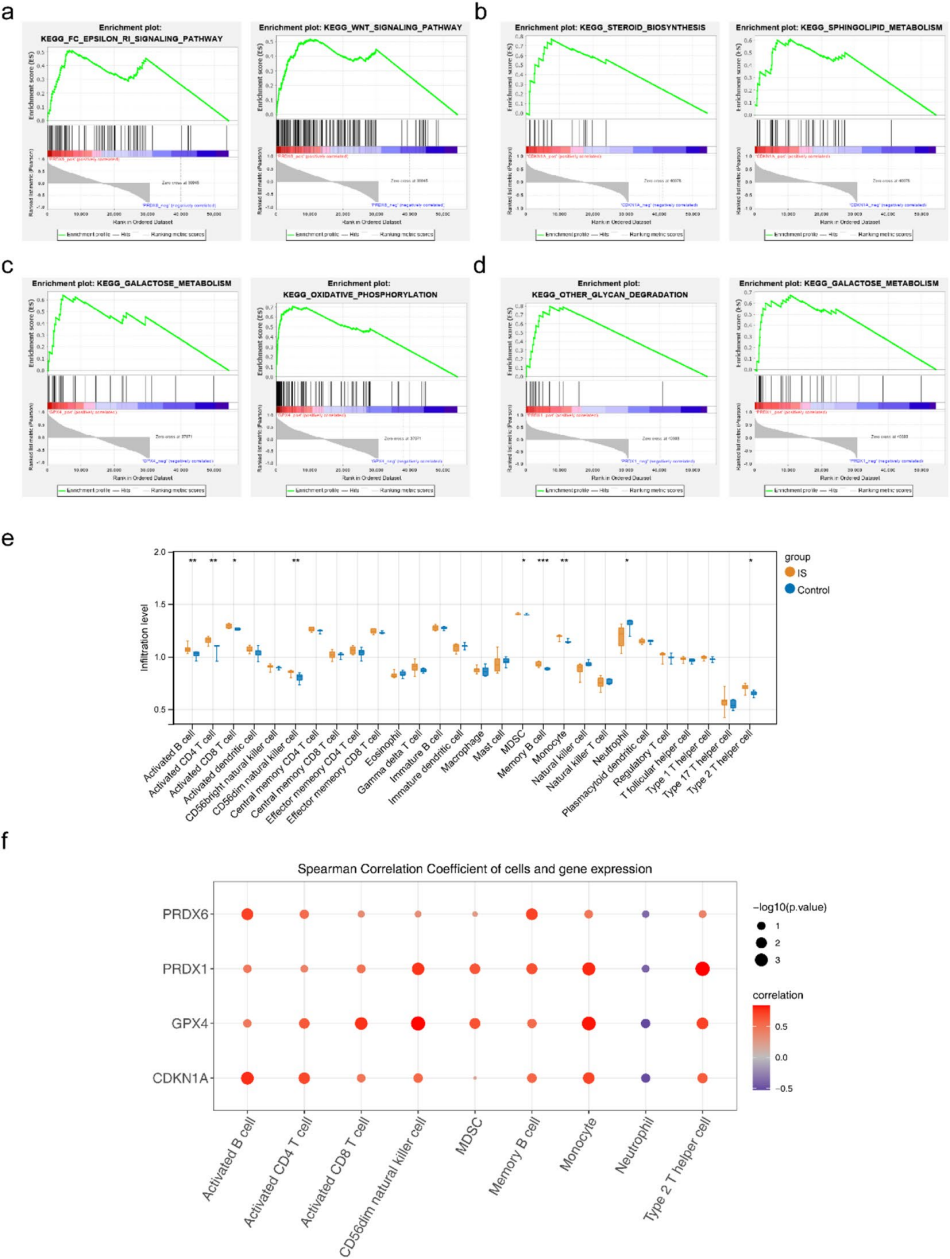
GSEA enrichment analysis of PRDX6 and immunocyte infiltration analysis. (**a**) enplot_KEGG_FC_EPSILON_RI and WNT_signaling pathway. (**b**) enplot_KEGG_steroid biosynthesis and sphingolipid metabolism. (**c**) enplot_KEGG_galactose metabolism and oxidative phosphorylation. (**d**) enplot_KEGG_other glycan degradation and galactose metabolism. (**e**) Box plot of 28 immune cell expressions between groups. (**f**) Bubble chart of correlation between biomarkers and differential immune cells.

According to KEGG functional enrichment analysis, PRDX6 was mainly enriched in the FC epsilon RI and WNT signaling pathways. CDKN1A is associated with steroid biosynthesis and sphingolipid metabolism. GPX4 is involved in galactose metabolism and oxidative phosphorylation. Besides, it was found to PRDX1 participated in glycan degradation and galactose metabolism (Fig. 5a–d, Supplementary Table 3). Furthermore, we performed immune infiltration analysis using the GSE122709dataset. The results showed that there were nine immune cells with significant differences (p < 0.05) between IS and control samples, including activated CD8 T cells, activated CD4 T cells, activated B cells, MDSC, CD56dim natural killer cells, neutrophils, type 2T helper cells, memory B cells, and monocytes (Fig. 5e). A strong positive correlation was observed between PRDX1 and type 2T helper cells, and GPX4 showed the strongest negative association with neutrophils (Fig. 5f, Supplementary Table 4).

### The construction of miRNA-mRNA-TF network and chemical drug prediction

**Figure 6:**
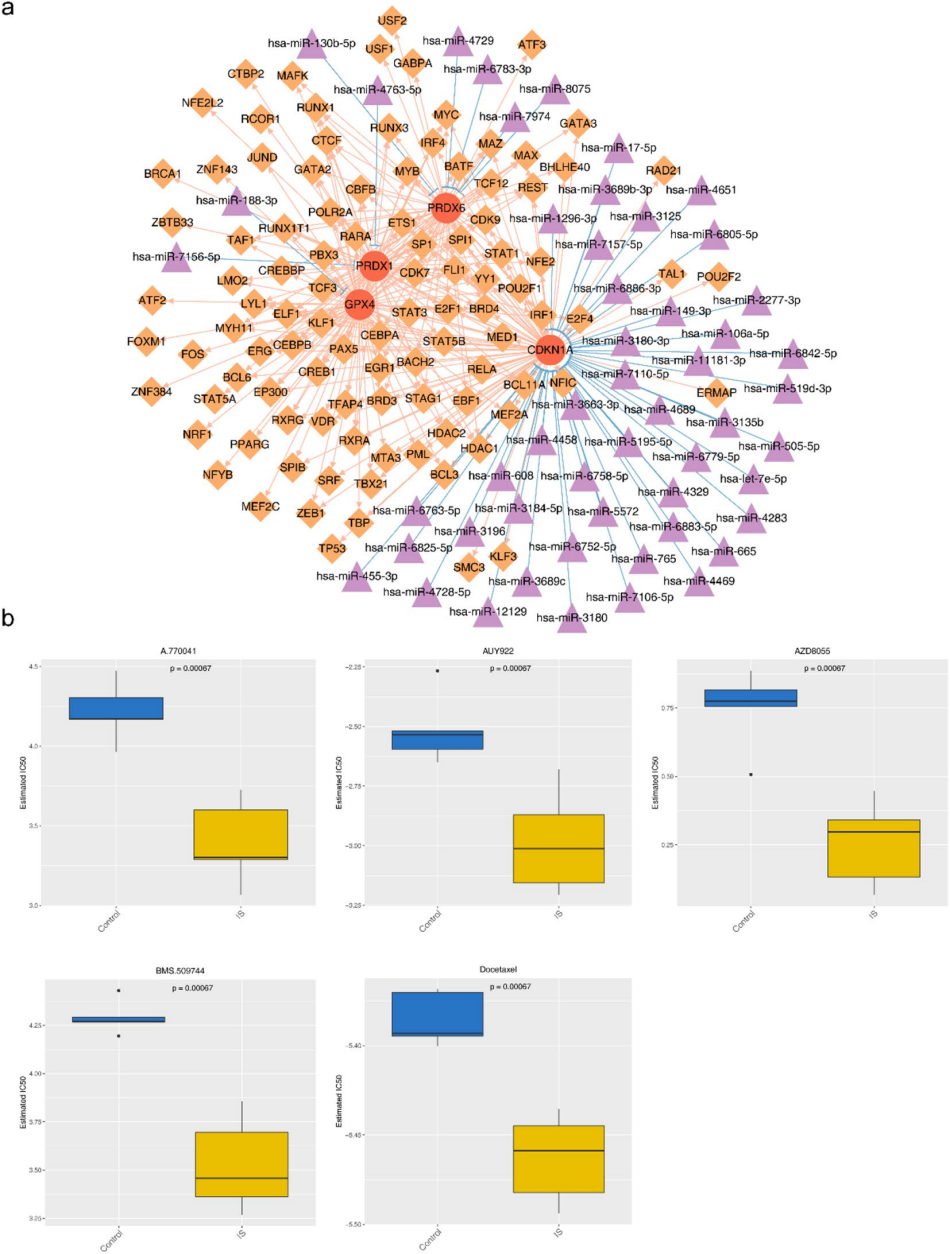
Construction of biomarker regulatory network and chemical drug prediction analysis. (**a**) Network diagram of miRNA-mRNA-TF regulatory mechanism. (**b**) IC_50_ Box Chart of A.770041, AUY922, AZD8055, BMS.509744, and Docetaxel Therapeutically Sensitive Drugs.

A total of 52 mRNA-miRNA pairs were screened, including 52 miRNAs and four mRNAs. Moreover, 260 mRNA-TF pairs were obtained, including 102 TFs and 4 mRNAs. There were 3747 miRNA-mRNA-TF regulatory pairs in the miRNA-mRNA-TF regulatory network, including hsa-miR-4469-CDKN1A-BACH2 and hsa-miR-188-3p-GPX4-ATF2 (Fig. 6a, Supplementary Table 5). In addition, there were significant differences in the therapeutic sensitivity of the 50 drugs in the IS samples, including A.770041, AUY922, AZD8055, BMS.509744, and docetaxel (Fig. 6b, Supplementary Table 6).

### The verification of qRT-PCR

**Figure 7:**
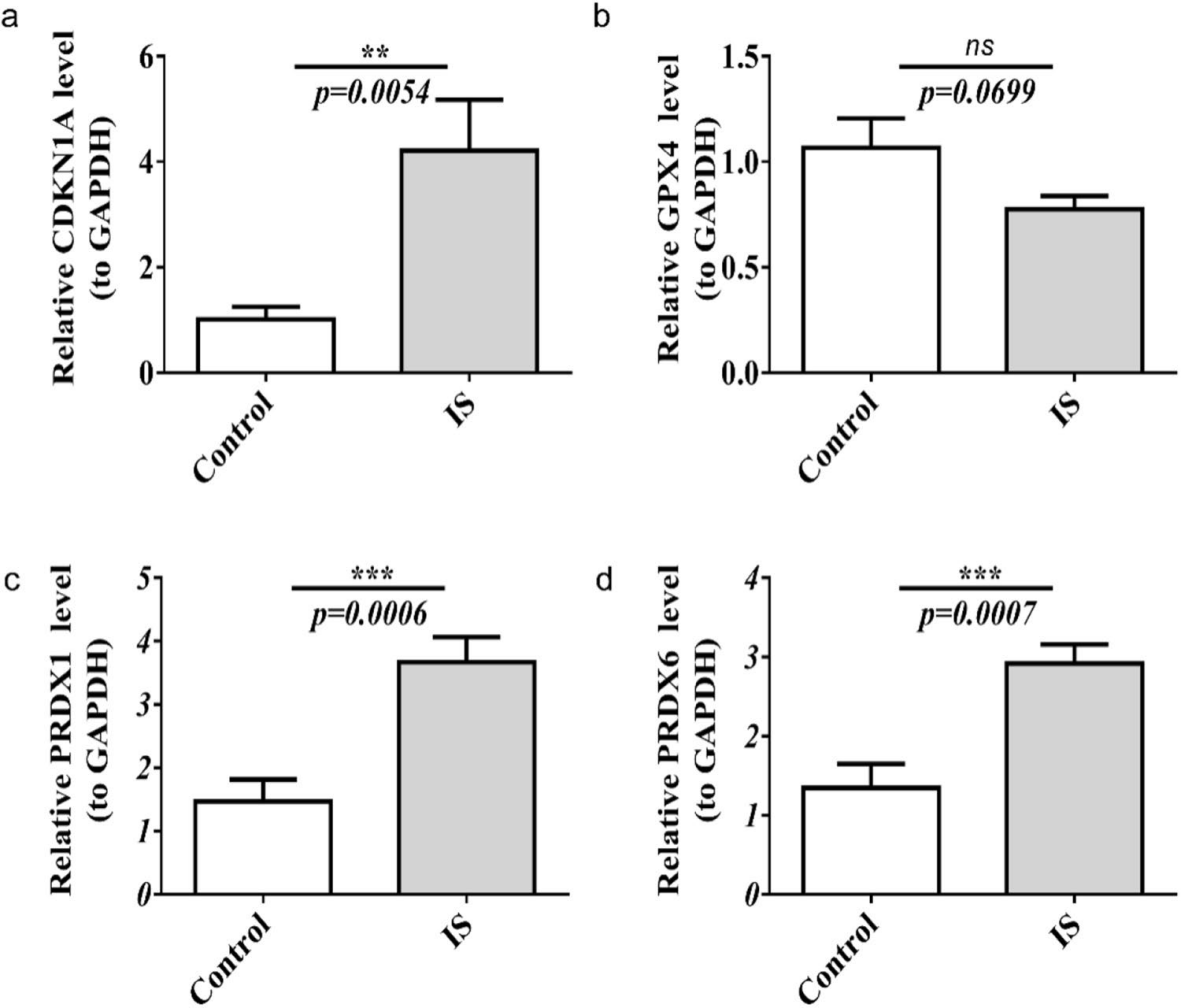
The relative mRNA expression level in patients with IS. (**a**–**d**) The relative CDKN1A, GPX4, PRDX1, and PRDX6 mRNA expression level in patients with IS. ns, not significant, ** p < 0.01, *** p < 0.001.

Based on the qRT-PCR results, CDKN1A, PRDX1, and PRDX6 were upregulated in IS samples, and the validation results were consistent with the above analysis (Fig. 7a–d).

## Discussion

Studies have found that stroke triggers neuronal ferrozoticism and that the use of siderotic inhibitors can reduce the degree of neuronal damage^24^. In IS, oxidative stress can lead to increased iron levels within the nerve cells and increased brain damage. Inhibits the activity of ACSL4 and lipoxygenase isotype 5 (LOX-5), thereby protecting cells from ferrozoticism^25^. The ROS scavengers ferrostatin-1 and liproxstatin-1 can specifically reduce ROS and inhibit ferroptosis^26^. Therefore, oxidative stress- and ferroptzosis-related genes play important roles in the occurrence and development of IS, and studying the relationship between oxidative stress and ferroptzosis-related genes can help determine the prognosis, efficacy, and therapeutic targets of IS patients.

In this study, four biomarkers, CDKN1A, GPX4, PRDX1, and PRDX6, were used to diagnose stroke and assess the treatment effects. Previous research has found that the antioxidant enzyme PRDX1 controls stroke-associated microglia in acute ischemic stroke^27^. CDKN1A/JUN is a robust and promising diagnostic biomarker for identifying patients with IS and may regulate ferroptosis during IS progression via the C9orf106/C9orf139-miR-22-3p-CDKN1A and GAS5-miR-139-5p/miR-429-JUN axes^28^. GPX4 can reduce peroxidized lipids; thus, inhibition of GPX4 or cystine import can induce ferroptosis^29^. Coenzyme Q10 (CoQ10) can reverse lipid peroxidation independently of GPX4; ferroptosis suppressor protein 1 (FSP1) can act as an oxidoreductase of CoQ10, shuttling reductants to the lipid bilayer plasma membrane to protect against peroxidation damage^29^. Phospholipase A2 of PRDX6 can improve cerebral ischemia/reperfusion inflammatory injury by reducing the expression of the inflammatory cytokines IL-1β, IL-17, and IL-23 and oxidative stress^30,31^. According to KEGG functional enrichment analysis, PRDX6 is mainly enriched in the FC epsilon ri and WNT signaling pathways, and the WNT signaling pathway plays an important role in the regulation of oxidative stress^32^. GPX4 is involved in galactose metabolism and oxidative phosphorylation^33^. Studies have found that glutathione peroxidase 4 (GPX4) uses glutathione as a substrate for redox reactions, reducing cellular lipid peroxides to their corresponding alcohols. Glutathione produces oxidized glutathione, thereby preventing cell death caused by the accumulation of lipid peroxides^34^. Recent studies have shown that GPX4 is also involved in ischemia–reperfusion-induced brain injury^35^. CDKN1A was also known as P21, when DNA damage occurs at cell telomeres, CDKN1A is able to cause cell cycle arrest and DNA repair by inhibiting the activity of cell cycle protein-dependent kinases, allowing cells to return to homeostasis^36^. CDKN1A, a regulator of ischemia–reperfusion injury, is associated with atherosclerosis and risk of myocardial infarction progression^37,38^. The myocardial infarction-related exosomes miR-208b and miR-208b regulate the growth of HUVECs by regulating CDKN1A expression^39^. Fan et al. suggested that CDKN1A could be a robust and promising diagnostic biomarker for IS by regulating ferroptosis during IS progression^28^. PRDX1 (peroxyregen 1) is a typical 2-cysteine (Cys) peroxyrein that catalyzes the reduction reaction and converts hydrogen peroxide (H_2_O_2_) to water as a multifunctional antioxidant^40^. The upregulation of PRDX1 in cells and tissues under oxidative stress is thought to be a cellular response to oxidative injury^40–42^. PRDX6 not only catalyzes the catalytic reduction of hydrogen peroxide and short-chain organic fatty acid peroxide activities of PRDX1, but also reduces phospholipid hydroperoxides, calcium-independent phospholipase A2 activity^43^, and lysophosphatidyltransferase activity^44^. PRDX6 repairs regenerated oxidized cell membranes^45,46^. Previous studies have shown that PRDX6 secreted by Schwann-like cells protects neurons against IS in rats via the PTEN/PI3K/AKT pathway^47^. Therefore, CDKN1A, GPX4, PRDX1, and PRDX6 are associated with ischemia–reperfusion-induced oxidative stress and ferroptosis. To observe the changes in the four biomarkers CDKN1A, GPX4, PRDX1, and PRDX6 in patients with ischemic stroke, we measured their expression of these four biomarkers in peripheral blood using RT-PCR. The results showed that CDKN1A, PRDX1, and PRDX6 expression levels were significantly increased, which was consistent with our study, suggesting that CDKN1A, PRDX1, and PRDX6 may be involved in the occurrence and development of ischemic stroke. Furthermore, in the training set GSE122709, the AUC values of all four biomarkers were above 0.9, indicating that the genes could perfectly distinguish between the IS and control samples. This superior performance might be attributable to the limited sample size, which could artificially enhance the discriminative precision of the biomarkers. To corroborate these findings, we assessed the efficacy of the model by using an independent validation set. Consistently, the biomarkers maintained high AUC values in this validation cohort, further substantiating the robustness of the model and its potential to be generalized across previously unseen data. These observations were consistent with those of previous studies^48–50^.

MAPK8 showed high connectivity in the PPI networks. Studies have shown that MAPK8 is also involved in the regulation of oxidative stress in tissues and cells^51,52^. In the PPI network, Cluster 1 contained 14 candidate mRNAs, including BECN1, MTOR, STAT3, MAPK1, DDIT3, CDKN1A, HMOX1, JUN, HSPB1, XBP1, KEAP1, SRC, ATG5, and SQSTM1. Recent studies have shown that the ATM-CHK2-Beclin 1 axis promotes autophagy to maintain ROS homeostasis under oxidative stress in a rat model of cerebral stroke^53^. Inhibition of PI3K/Akt/mTOR signaling by NDRG2 contributes to neuronal apoptosis and autophagy in IS^54^. Neuronal STAT3/HIF-1α/PTRF axis-mediated bioenergetics can exacerbate cerebral ischemia–reperfusion injury via PLA2G4A^55^. The β-Arrestin-2-ERK1/2 cPLA2α axis mediates TLR4 signaling and influences the induction of eicosanoids in the ischemic brain^56^. Activation of ATF4-DDIT3-mediated ER stress promotes proliferation and protects cortical neural stem cells in vitro^57^. The expression of miR-20a is upregulated by stroke serum, which promotes MSC proliferation by regulating the cell cycle inhibitor p21 CDKN1A^58^. heme oxygenase 1 (HMOX1)-mediated neurogenesis after permanent IS in mice^59^. The absentia homolog 1/Jun N-terminal kinase pathway can reduce oxidative stress and mitochondrial damage in rats with cerebral ischemia–reperfusion injury^60^. HspBs, HspB1, and HspB5 may be most important in the neuronal stress response to ischemia/reperfusion injury in the brain and may be involved in neuroprotection^61^. Downregulation of XBP-1 can rescue pyroptosis induced by cerebral ischemia/reperfusion injury through the NLRP3/Caspase-1/GSDMD axis^62^. The Keap1-Nrf2/ARE signaling pathway is activated by butylphthalide during IS treatment^63^. Inhibition of the Src-PP2B-mTOR pathway activity could improve neuronal ischemic injury^64^. ATG5 knockdown attenuates ischemia‒reperfusion injury by reducing excessive autophagy-induced ferroptosis^65^. SQSTM1 attenuates oxygen–glucose deprivation/re-oxygenation-induced neuronal injury in vitro^66^. Therefore, these intersecting mRNAs may play important roles in the occurrence and development of IS.

Stroke-prone immunosuppression (SIID) occurs in both experimental models and clinical cases. SIID is characterized by lymphopenia, upregulation of anti-inflammatory cytokines, and splenomegaly atrophy^67^. Other factors that influence SIID include glucocorticoids, acetylcholine, epinephrine, and norepinephrine^68^. However, the mechanisms by which stroke causes cellular immune dysfunction remain unclear. In 1979, Czlonkowska et al.^69^ found a decrease in the total number of peripheral blood lymphocytes and a concomitant decrease in T lymphocytes in stroke patients, suggesting that it is related to stress. Berczi^70^ suggested that cerebral infarction causes increased functional activity of the hypothalamic–pituitary–adrenal axis, producing large amounts of adrenocorticotropic hormones, which decreases the number of CD3+ and CD4+ T lymphocytes, thus suppressing the immunity of the body. Fiorina^71^ observed that cerebral infarction was associated with a decrease in plasma melatonin (MT) levels, but not adrenocortical hormone levels. Campanella et al.^72^ used flow cytometry to detect a significant increase in lymphocytes after cell isolation in cerebellar tissue from cerebral infarction, further suggesting that the decrease in peripheral blood T-lymphocytes may be due to a large number entering the center. In this study, immune infiltration analysis showed that nine immune cell types were significantly different between the IS and control groups: activated CD8 T cells, activated CD4 T cells, activated B cells, MDSC, CD56dim natural killer cells, neutrophils, type 2T helper cells, memory B cells, and monocytes. However, the mechanism of inhibition in previous studies remains unclear and requires further investigation. Further studies found that PRDX1 had the strongest positive correlation with type 2T helper cells, consistent with previous studies^73,74^, and GPX4 had the strongest negative correlation with neutrophils, consistent with previous studies^75,76^.

miRNAs play an important role in IS development^77^. In this study, 3747 miRNA-mRNA-TF regulatory pairs were identified in the miRNA-mRNA-TF regulatory network, including hsa-miR-4469-CDKN1A-BACH2 and hsa-miR-188-3p-GPX4-ATF2. The current study found that the miR-188-3p/GPX4 Signaling axis is involved in the improvement of germacrone-mediated diabetic nephropathy by regulating ferroptosis^78^, and miR-4469-CDKN1A is only involved in the development of multiple cancers^79,80^. In addition, there were significant differences in the therapeutic sensitivities of the 50 drugs in the IS samples, including A.770041, AUY922, AZD8055, BMS.509744, and docetaxel. A previous study found that A-770041 could reverse paclitaxel and doxorubicin resistance in osteosarcoma cells and prevent organ allograft rejection^81,82^. Inhibition of Hsp90 by AUY922 is an effective strategy for the treatment of myxoid liposarcoma^83^. Moreover, AZD8055 induces autophagy and AMPK activation-related cell death in hepatocellular carcinoma^84^. BMS-509744 is a selective inhibitor of interleukin-2-inducible T-cell kinase, and topical application can ameliorate imiquimod-induced skin inflammation in mice^85^. Docetaxel can remodel the immune microenvironment of prostate cancer and enhance immunotherapy based on checkpoint inhibitors^86^. However, none of these drugs has been used in the treatment and research of IS, which needs to be confirmed in further studies. These results provide a theoretical basis for the treatment, evaluation, and research on the efficacy of IS.

This study identified a diagnostic association between oxidative stress-ferrozosis and IS genes, and drug prediction analysis was performed to provide a new reference for the diagnosis and treatment of patients with IS. However, this study had some limitations.

Initially, the identification of biomarkers was constrained by the limited scope of clinical samples available in public databases, which necessitates the critical expansion of the sample size for robust analysis. Furthermore, while the diagnostic utility of these biomarkers has been appraised using ROC curves, it is imperative to recognize that this approach represents only a single assessment dimension. A more comprehensive and substantiated basis for clinical decision making involves integrating clinical data with multiple evaluative metrics. Consequently, the purported clinical diagnostic merit of the four identified biomarkers warrants further validation through extensive validation using large-scale clinical samples. Furthermore, it is necessary to compare these four biomarkers with standard IS biomarkers to evaluate their value in the prognosis and treatment effectiveness of patients with IS. Correspondingly, the efficacy and cost-effectiveness of the resultant targeted therapies derived from analytical methods must be evaluated thoroughly. Finally, future research endeavors should prioritize elucidating the molecular mechanisms underlying these biomarkers by utilizing well-constructed animal or cellular models.

## Conclusions

Oxidative stress can increase the intracellular iron concentration, and iron-catalyzed Fenton and Haber–Weiss reactions can produce a large amount of free radicals, further exacerbating the oxidative stress response. During ferroptosis, iron reacts with unsaturated fatty acids in the cell membrane to form hydroxyl radicals, which can cause membrane lipid peroxidation and induce ferroptosis. Therefore, the relationship between oxidative stress and iron death is very close, and the two mutually promote and affect each other, respectively. In this study, we identified biomarkers associated with oxidative stress and ferroptosis using bioinformatics and achieved good classification results, which can provide new directions and methods for the early diagnosis and treatment of IS. We also analyzed the biological functions of biomarkers and revealed the important roles of oxidative stress and iron death in IS pathogenesis, providing new clues and theoretical support for a deeper understanding of IS pathogenesis. We believe that our research direction and results make our research novel and unique with academic value.

### Supplementary Information

Supplementary Legends.

Supplementary Table 1.

Supplementary Table 2.

Supplementary Table 3.

Supplementary Table 4.

Supplementary Table 5.

Supplementary Table 6.

## Data Availability

All other raw data were accessed by contacting the corresponding author, if any qualified researcher required them.

## Abbreviations

IS: Ischemic stroke
SIID: Stroke-prone immunosuppression
WGCNA: Weighted gene co-expression network analysis
GEO: Gene expression omnibus
GSEA: Gene Set Enrichment Analysis
DEGs: Differentially expressed genes
PRDX1: Peroxyregen 1
H_2_O_2_: Hydrogen peroxide
MT: Melatonin
HMOX1: Heme oxygenase 1
PTMs: Protein post-translational modifications
qRT-PCR: Quantitative real-time polymerase chain reaction
GPX4: Glutathione peroxidase 4
LOX-5: Lipoxygenase isotype 5
OS: Oxidative stress
ORGs: Oxidative stress-related genes
FRGs: Ferroptosis-related genes
DEmRNA: Differentially expressed mRNA
ORGs: Oxidative stress-related genes
PPI: Protein–protein interaction
OS: Oxidative stress
ROS: Reactive oxygen species
