# Supplementary material for: Analysis and identification of oxidative stress-ferroptosis related biomarkers in ischemic stroke
Source: Sci Rep. 2024 Feb 15;14:3803. doi: 10.1038/s41598-024-54555-2 (PMC10869843; doi:10.1038/s41598-024-54555-2)
Supplement: Supplementary file 1 — Supplementary Legends. [file 41598_2024_54555_MOESM1_ESM.docx]

**Supplementary information**

**Supplementary Table 1** GO terms enriched in 30 intersection mRNAs.

**Supplementary Table 2** KEGG terms enriched in 30 intersection mRNAs.

**Supplementary Table 3** The results of GSEA for four biomarkers.

**Supplementary Table 4** The relevance of biomarkers and differential immune cells.

**Supplementary Table 5** The interaction of miRNA-mRNA-TF regulatory network.

**Supplementary Table 6** The list of drugs with different sensitivity.
